# Effect of a Multimodal Movement Intervention in Patients With Neurogenic Claudication Based on Lumbar Spinal Stenosis and/or Degenerative Spondylolisthesis—A Pilot Study

**DOI:** 10.3389/fmed.2020.540070

**Published:** 2020-12-08

**Authors:** Kim-Charline Broscheid, Tom Behrendt, Dennis Hamacher, Svantje Böker, Tabea Gagelmann, Christian Schmidt, Christina Caspari, Katharina Meiler, Andre Napiontek, Jörg Franke, Lutz Schega

**Affiliations:** ^1^Department Human Science, Sport Science, Institute III, Otto von Guericke University Magdeburg, Magdeburg, Germany; ^2^Department of Orthopaedic Surgery, Klinikum Magdeburg, Magdeburg, Germany; ^3^Association for Health, Exercise and Sport, Magdeburg, Germany

**Keywords:** lumbar spinal stenosis, degenerative spondylolisthesis, chronic low-back pain, spinal cord claudication, gait variability, risk of falling, functional mobility, the timed “up & go” test

## Abstract

Chronic low-back pain is a major individual, social, and economic burden. The impairment ranges from deterioration of gait, limited mobility, to psychosocial distress. Due to this complexity, the demand for multimodal treatments is huge. Our purpose is to compare the effects of a multimodal movement intervention (MI) (coordinative–cognitive exercises and dancing program) with standard physical therapy (PT) on gait, physical function, and quality of life in patients with lumbar spinal stenosis (LSS). The study design is based on a 6-week intervention with a two (group: MI/PT) by two (measurement time points: pre-/post-test) parallel group design with random assignment. Twenty-four subjects (18 female/6 male, 70.8 ± 10.6 years old) diagnosed with LSS were included and randomly allocated to the MI or PT group. The primary outcomes are minimum toe clearance (MTC) and double step length (DSL) variability and the Timed “Up & Go” test (TUG). Secondary outcomes are the Brief Pain Inventory, the short Fall Efficacy Scale–International (sFES-I), and the Oswestry Disability Index. Nine subjects for each group could be analyzed. The MTC variability revealed a significant between-group difference in the posttest (*p* = 0.008) showing a lower MTC variability for the MI compared to the PT group. The MI group displayed an improved TUG (*p* = 0.031) and a reduced sFES-I (*p* = 0.044). The decreased MTC variability and fear of falling as well as the improved functional mobility may contribute to a reduced risk of falling. For the subsequent study, further kinematic and cognitive parameters should be analyzed, and the number of participants has to be increased.

**Clinical Trial Registration:** German Clinical Trial Register (ID: DRKS00021026/URL: https://www.drks.de/drks_web/navigate.do?navigationId=trial.HTML&TRIAL_ID=DRKS00021026).

## Introduction

Over 500 million people worldwide suffer from low-back pain (LBP) and the associated health restrictions, regardless of gender, or economic status (1, 2). Patients with chronic LBP, which is defined as pain in the lumbar spine for at least 3 month, are a small but economically relevant subgroup of LBP patients in the healthcare system (3, 4). Closely related to chronic LBP are degenerative changes resulting in spondylolisthesis and lumbar spinal stenosis (LSS) contributing to neurogenic claudication (5, 6). The resulting constraints are not only physical loss of function but are also perceived on a psychosocial level, resulting in reduced quality of life, and life expectancy (7, 8). Additionally, the pain is often accompanied by insufficient muscular stabilization and poor posture (9, 10). All these factors lead to a change in gait pattern (11, 12), which lead to an increased risk of falling. Several studies indicate that people with chronic LBP have a higher prevalence of falls than people of comparable ages (13–15). This can lead to severe health consequences (16) and is a personal, social, and economic burden.

Due to the described manifold biosocial effects and since most of the affected people cannot attribute the pain to a specific nociceptive event (2), a purely biomedical addressing of the complaints is not suitable for many patients. Here, the concept of the biopsychosocial model helps to transform medically defined diseases into complex symptom profiles of patients and captures the various limitations and complaints (17). In this context, multimodal approaches are essential to treat chronic LBP and the associated symptoms successfully (2) One approach is to complement classical exercises for muscle stabilization with cognitive training. Cognitive training already plays an increasingly important role in multimodal rehabilitation concepts to strengthen physical and mental resources avoiding disease- or pain-related limitations (e.g., reduced mobility) (18–20). Furthermore, it is well-known that the performance capability of certain cognitive functions, in particular attention, executive functions, and working memory, are strongly associated with the extent of gait control (e.g., gait variability, gait velocity) (21–24). Evidence from previous studies suggests that multimodal exercise interventions integrating coordinative (motor) and cognitive elements can have superior effects on brain plasticity and cognitive functions compared to single modality interventions (25–28). This may be due to supplementary indirect mechanisms such as improved cardiovascular fitness, muscle mass as well as muscle strength, and direct mechanisms such as task-specific neuroplasticity (29).

Therefore, a dance training consisting of complex sensorimotor, rhythmic, and physical activity integrating multiple cognitive and social elements (30–32) combined with a coordinative–cognitive demanding training could have superior effects on designated cognitive functions, leading to a decreased gait variability. Several studies already applied dancing interventions in elderly with and without mild cognitive impairment, indicating an improvement in cognitive functions (25, 33, 34). These multiple improvements especially the reduction in gait variability could lead to a reduced risk of trip-related falls in people with chronic LBP.

For this reason, the objective of this study is to evaluate the beneficial effects of a multimodal intervention (MI) composed of a coordinative–cognitive training and a dancing program in contrast to standard physical therapy (PT) on the risk of falling in patients with chronic LBP, neurogenic claudication in LSS and/or degenerative spondylolisthesis. We expect that the gait variability, functional mobility, perceived pain, and fear of falling improve more by the MI than by PT.

## Materials and Methods

### Participants

Thirty-two subjects (24 female/8 male) were recruited by a newsletter announcement distributed in two different clinical institutions in Magdeburg from January to March 2019. All subjects were at least 60 years old, had LSS (decompression laminectomy), and showed clinical relevant evidence for chronic LBP and neurogenic claudication and/or degenerative spondylolisthesis diagnosed by medical professionals. The exclusion criteria were musculoskeletal disease, neurological disorders, congenital spinal malformations, and spinal surgical interventions (<2 years ago). Twenty-four subjects (18 female/6 male), on average, 70.8 ± 10.6 years old, could be included. Due to a probably higher dropout rate in the MI group, we have initially assigned 14 participants to the MI and 10 to the PT group.

### Trial Design and Settings

The present study was designed as a 6-week pilot intervention study with a two (group: MI/PT) by two (measurement time points: pre-/post-test) parallel group design with random assignment. The research protocol was in accordance with the principles of the Declaration of Helsinki and was approved by the ethics committee of the Otto von Guericke University (OvGU) Magdeburg (Germany) (registration number: 182/18). At both time points (pre-/post-test), the subjects were invited to the Department of Orthopedic Surgery at Klinikum Magdeburg (MVZ, Germany). At the first meeting, the participants were informed in detail about the study purpose, and written informed consent was obtained. Thereafter, an interview was conducted to assess the course of the disease, comorbidities and the location as well as the current intensity of pain related to the chronic LBP. Additionally, the Timed “Up & Go” test (TUG) (35) was performed. The time that a participant needed to get up from a chair, walk 3 m, turn 180°, walk back to the chair, and sit down again was recorded. The subjects were instructed to perform the test fast but safely. Subsequently, they were asked to complete three questionnaires. The first questionnaire, the Brief Pain Inventory (BPI/German version) (36), asks subjects to rate their pain intensity and their pain interference with domains in everyday living (e.g., walking, working, mood, sleep quality) on a scale ranging from 0 to 10 (0 = “no pain”/“does not interfere;” 10 = “pain as bad as you can imagine”/“Completely interferes”). Furthermore, the short Falls Efficacy Scale–International (sFES-I) (37) was used to determine the fear of falling. It is scored with a minimum of 7 (no concern about falling) to a maximum of 28 (severe concern about falling). Finally, the modified version of the Oswestry Disability Index (ODI, version 2.1) (38) was applied, which consists of 10 items to assess participants' back-pain-related disabilities in performing activities of daily living (e.g., personal care, lifting, walking, sitting, standing, sleeping, sex life, social life, traveling, working). Each item consisted of six statements, which are scored on a scale from 0 to 5 (0 = least amount of disability; 5 = most sever disability). The score of all items were summed and multiplied by 2 to obtain the index (range from 0 = no disability to 100 = maximum disability).

Following the medical interview, the acquisition of gait kinematics was also carried out at the MVZ in order to assess gait variability. The patients had to walk back and forth a distance of 12 m over 2 min at their preferred walking speed. The first and last 2.5 m of each walking-track were excluded from data analyses to deduct the deceleration and acceleration phases. The gait kinematics were captured with three inertial measurement units (sampling rate, 120 Hz) (MTw, Xsens Technologies B.V., Netherlands) fixed dorsally at each foot and one at the upper body (sternum). The gait protocol and the calculation procedure are based on the recommendations from Hamacher et al. (39). The parameters of interest were the double step length (DSL) variability and minimum toe clearance (MTC) variability. Before and after the session, the participants were asked about their state of pain using the Borg scale (40).

For both groups, adequate and medically secured treatment of pain was ensured by the intake of oral pain medications in accordance with WHO standards (41) and was not modified during the study. After the pretesting, the participants were randomly allocated to either the MI or the PT group by using the block randomization method (42).

### Intervention

The MI was offered twice a week for 60 min over 6 weeks at the gym of the OvGU. The exercise sessions of the MI group consisted of three different parts: (1) 10 min coordinative–cognitive training, (2) 40 min dancing program, and (3) 10 min cool-down. The coordinative–cognitive training included exercises that refer to the performance of motor–cognitive dual-task while simultaneously processing external information. Motor–cognitive dual-task exercises are defined as exercises where both motor task (e.g., walking) and cognitive task (e.g., counting backwards in series of 7) are performed at the same time. Therefore, the focus of these exercises was to perform a cognitively demanding task in addition to motor activities dictated by external information [e.g., solving an arithmetical task while catching a ball and the color determines how the ball is caught (e.g., left or right hand)]. The degree of complexity of these exercises was continuously adjusted so that the participants could not master the task to perfection. The dancing program consisted of different dancing choreographies including single and couple dance. The subjects had to learn, memorize, and access new moving patterns and step sequences under various physical and coordinative demands (e.g., physical strain, time pressure, precision pressure, complexity pressure). In addition, subjects were trained to couple body movements regarding different rhythms. In order to maintain a high level of coordinative demand, the complexity of the choreography was successively increased over the course of the intervention. The cool-down mainly consisted of relaxing and stretching exercises. The PT group received a standard prescription for standard physical therapy (general exercise therapy and motor control exercises) three times a week for 30 min. Each exercise session was supervised by experienced instructors or professional therapists, respectively. This applies to both the MI and PT group.

### Outcome Measures

The primary endpoint consisted of the variability of the DSL (43) and the MTC (44) as sensitive parameters for both a deterioration of gait and increase in risk of falling. As clinically relevant primary endpoint, the TUG was measured as an indicator for the functional mobility (35).

The secondary outcomes of the study consisted of pain intensity and interference with activities of daily living, fear of falling, and pain-related disabilities, as measured through BPI, sFES-I, and ODI scores, respectively.

### Statistical Analysis

Due to the low number of subjects that could be included in the analysis, we used non-parametric tests for all statistical analyses. Group differences in age, height, weight, body mass index (BMI), and changes in the outcome parameters (TUG, DSL variability, MTC variability, sFES-I, BPI, and ODI) were compared using the Mann–Whitney *U*-test. Changes between measurement time points within a group were verified using the Wilcoxon test (time effect). Outcome parameters are presented in median (25th/75th percentile). All statistical analyses were performed using SPSS Statistics 25 (IBM, Inc., Chicago Illinois) for Microsoft Windows. The level of significance was set to an alpha level of 5%.

## Results

**Figure 1:**
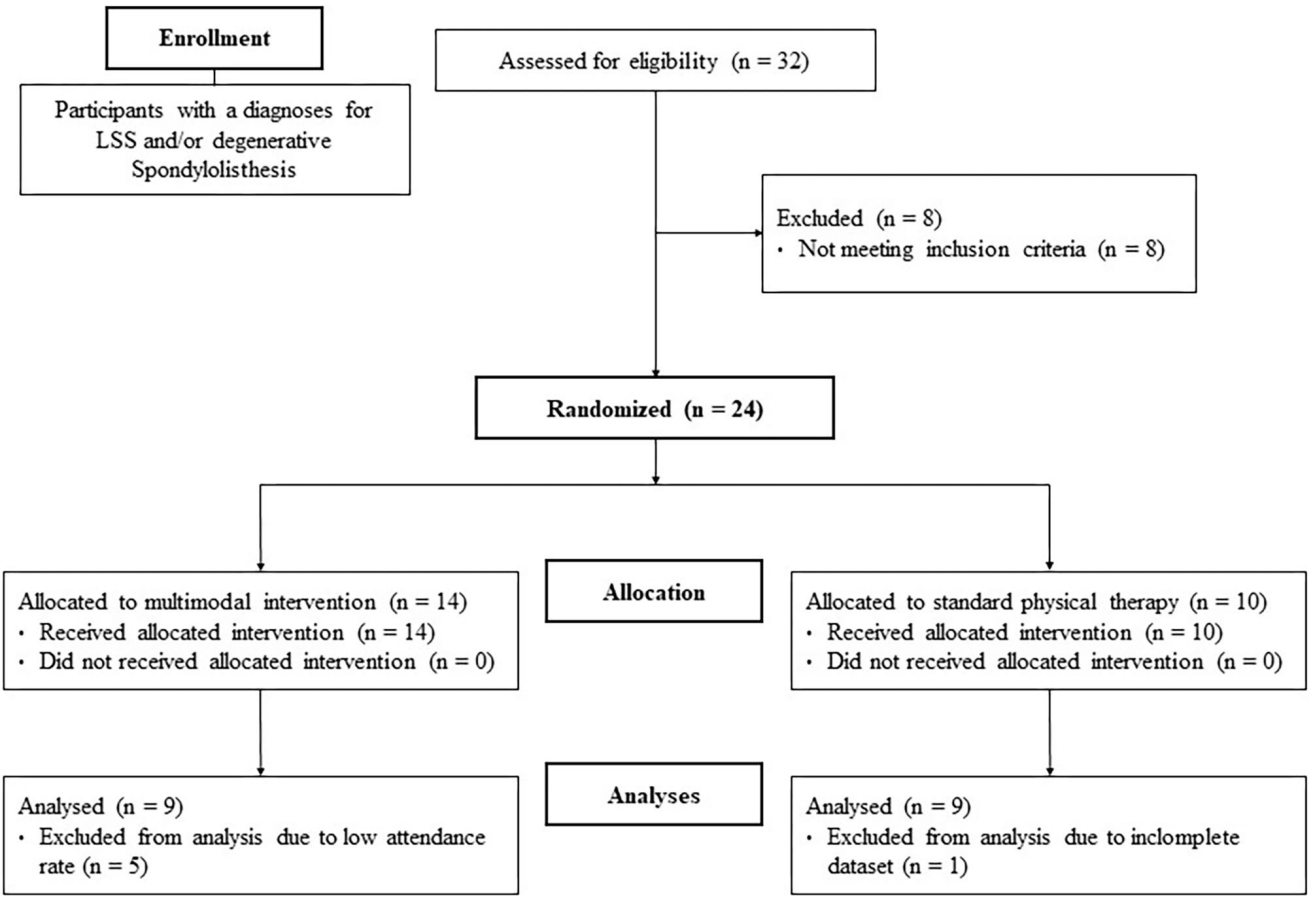
Flow chart of the study design.

The data of five subjects had to be excluded from the MI group due to a low attendance rate (<80%). The lack of participation in the intervention was explained by the participants with personal commitments. Additionally, one subject from the PT group was removed based on an incomplete dataset. Finally, the data of nine subjects for each group (MI/PT) was analyzed (see flowchart Figure 1). At baseline, the analysis of age, height, weight, and BMI revealed no significant differences between groups (see Table 1).

**Table 1:** Means (M) and standard deviations (SD) of age, height, weight, and body mass index (BMI) of the remaining subjects at baseline (*N* = 18).

**Group**	**MI Group^**a**^**	**PT Group^**a**^**
*N* (female/male)	9 (6/3)	9 (7/2)
Age (years)	75.0 (6.2)	68.0 (15.0)
Height (m)	166.8 (5.2)	164.7 (10.2)
Weight (kg)	80.6 (13.5)	76.4 (15.1)
BMI (kg/m^2^)	29.0 (5.2)	28.3 (5.4)

a *Subjects remaining after dropout due to low attendance rate and incomplete dataset*.

*MI, multimodal intervention; PT, standard physical therapy*.

Regarding the DSL and MTC variability, we did not find statistically significant differences between groups in the pretest (*Z* = −1.015, *p* = 0.340 and *Z* = −1.457, *p* = 0.161, respectively). The between-group difference concerning the DSL was also not statistically significant in the posttest (*Z* = −0.397, *p* = 0.730). The MTC variability revealed a significant between-group difference (*Z* = −2.605, *p* = 0.008), indicating that subjects of the MI group had a lower MTC variability after the intervention compared to the PT group (see Table 2). No statistically significant time effects occurred for both groups. However, a tendency (0.100 > *p* > 0.050) could be observed for the MI group, suggesting that these subjects reduced their variability of DSL and MTC in the posttest (MI: −20.92 and −7.56%, respectively; PT, −13.63 and −0.15%, respectively; see time effects in Table 2).

**Table 2:** Medians and 25th/75th percentiles of double step length (DSL) variability, minimum toe clearance (MTC) variability, short Falls Efficacy Scale–International (sFES-I), Brief Pain Inventory (BPI), Oswestry Disability Index (ODI), and Timed “Up & Go” test (TUG) for the multimodal intervention (MI), and standard physical therapy (PT) group in pre- and posttest.

	**MI Group**		**Time effect**	**PT Group**		**Time effect**
	***Pre***	***Post***		***Pre***	***Post***	
**Gait variability**						
DSL variability (cm)	9.17 (6.49/13.17)	6.16 (2.63/8.27)	*Z* = −1.718 *p* = 0.086	6.81 (3.07/10.57)	5.91 (1.90/9.20)	*Z* = −1.125 *p* = 0.260
MTC variability (mm)	6.21 (4.69/7.20)	5.74^*^ (4.35/6.43)	*Z* = −1.836 *p* = 0.066	9.08 (5.17/11.96)	8.97 (6.44/10.87)	*Z* = 0.178 *p* = 0.859
**Questionnaires**						
sFES-I	12.0 (9.5/14.0)	9.0 (8.0/13.5)	*Z* = −2.014 *p* = 0.044	11.0 (10.0/13.0)	10.0 (8.0/15.5)	*Z* = −0.494 *p* = 0.621
BPI	31.0 (22.5/43.0)	30 (23.5/51.0)	*Z* = −0.297 *p* = 0.766	18.0 (12.0/44.5)	33.0 (15.5/48.5)	*Z* = −1.304 *p* = 0.192
ODI	15 (8.0/16.5)	12.5 (7.5/17.3)	*Z* = 0.108 *p* = 0.914	12 (7.8/15.8)	12.5 (9.0/21.3)	*Z* = −1.086 *p* = 0.277
**Physical performance**						
TUG (s)	9.09 (8.83/14.65)	9.67 (7.74/13.23)	*Z* = −2.192 *p* = 0.028	10.43 (7.47/13.24)	10.38 (8.39/13.13)	*Z* = −0.889 *p* = 0.374

* *Group effect: significant different from physiotherapy group at posttest (statistical significant: p ≤ 0.05)*.

**Figure 2:**
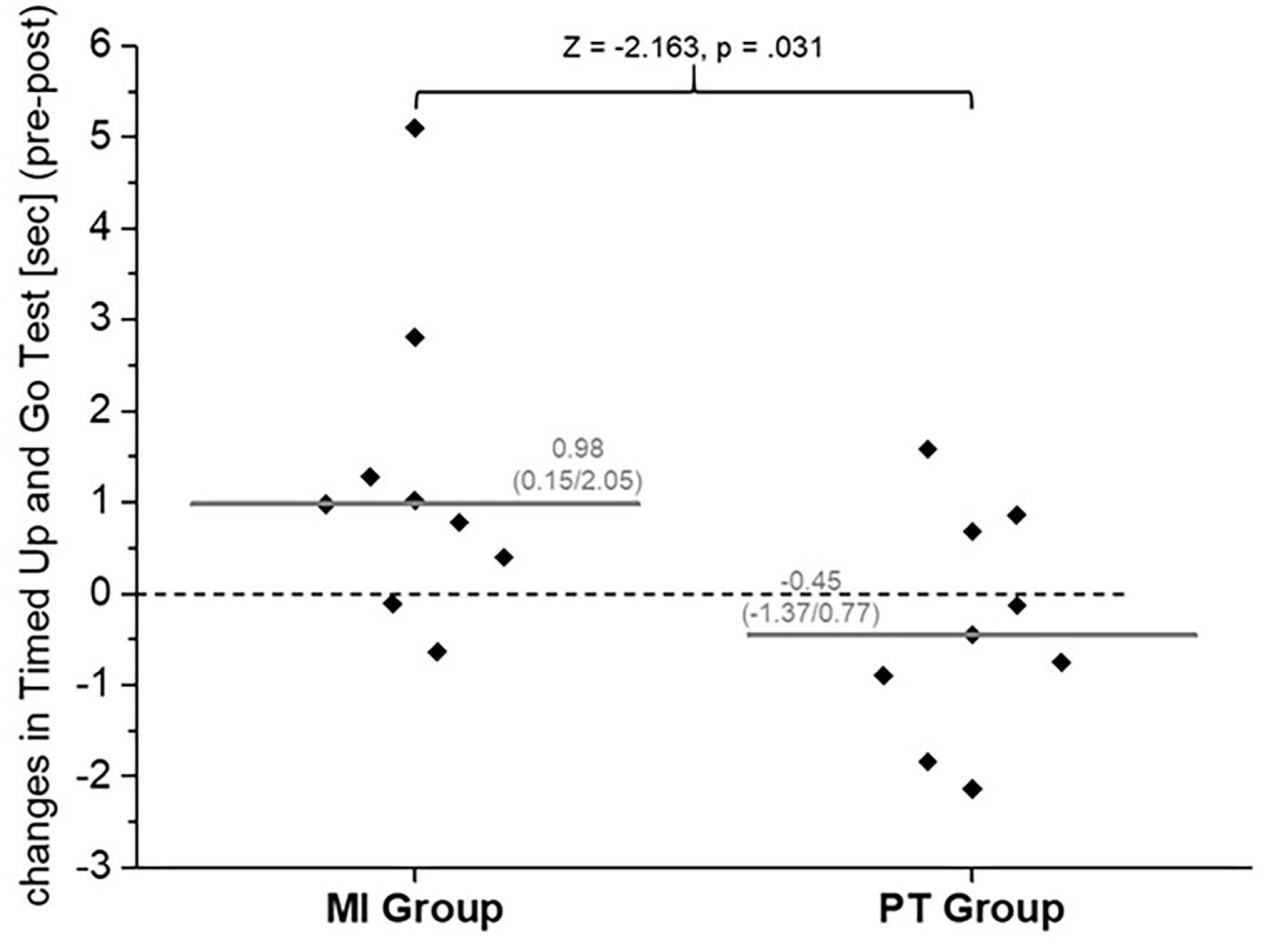
Median [gray line, median (25th/75th percentile)] and intra-individual (gray squares) changes in the Timed Up and Go Test [pre-/post-test (s)] for the multimodal intervention (MI) group and standard physical therapy (PT) group.

With respect to the TUG, we could not show statistically significant differences between groups in pre- or posttest (*Z* = −0.662, *p* = 0.546 and *Z* = −0.530, *p* = 0.605, respectively). For the MI group, we observed a significant time effect (see time effect in Table 2). Considering the differences from pre- to post-test within each group, it can be stated that the participants of the MI group reduced their time taken to complete the TUG by almost 1 s, whereas the PT group did not (see Figure 2).

For the s-FES-I, the BPI, and the ODI, we could not display any statistically significant differences between the MI and PT group in the pretests [sFES-I: 12.0 (9.5/14.0) and 11.0 (10.0/13.0), *Z* = −0.358, *p* = 0.730 and *Z* = −0.845, *p* = 0.436, respectively; BPI: 31.0 (22.5/43.0) and 18.0 (12.0/44.5), *Z* = −1.016, *p* = 0.340 and *Z* = −0.354, *p* = 0.730, respectively; ODI: 15.0 (8.0/16.5) and 12.0 (7.8/15.8), respectively, *Z* = −0.246, *p* = 0.842 and *Z* = −0.357, *p* = 0.762, respectively]. Furthermore, no significant between-group differences could be observed after 6 weeks of intervention (sFES-I: *Z* = −0.845, *p* = 0.436; BPI: *Z* = −0.354, *p* = 0.730; ODI: *Z* = −0.357, *p* = 0.762). Regarding the sFES-I, we could observe a significant time effect, demonstrating that the fear of falling is reduced after 6 weeks of intervention in the MI group [−3.0 (0.0/−3.5)]. In the PT group, however, the sFES-I remained unchanged over time [−1.0 (2.0/−3.5)]. Regarding BPI and ODI, we could not determine any time effects in either group (see Table 2).

## Discussion

This pilot study has revealed first tendencies that a multimodal approach consisting of a coordinative–cognitive training and dancing program reduces gait variability (not significant) and the fear of falling and improves the level of functional mobility more than the standard PT. These results are congruent with other equivalent studies. Cruz-Díaz et al. (45) compared a 6-week Pilates intervention to standard PT in older women with chronic LBP. They also demonstrated a reduction in the fear of falling (sFES-I) and an improvement in functional mobility (TUG). Against our expectations, the perception of pain (BPI) and the pain-related disabilities in performing activities of daily living (ODI) have remained unchanged for both groups in our pilot study. Other studies applying a belly dance program (46), exercise therapy (47), or a videogame-based exercise program (48) were able to demonstrate reduced pain perception in chronic LBP patients.

Moreover, we could observe a group effect, suggesting that the MTC variability was significantly lower in the MI compared to the PI group at the end of the intervention (see Table 2). The DSL variability remained unchanged. Additionally, there was a trend toward a time effect, indicating that gait variability (DSL and MTC) was slightly reduced in subjects who had completed the MI. We can only speculate that this trend could become significant with a higher number of subjects. These results are in line with previous studies investigating the effects of a dancing program on parameters of gait variability. Hamacher et al. (25) showed that a 6-month dancing program (twice a week at 90 min) lowers MTC variability in dual-task walking (thinking while walking) to a higher extent than a conventional strength–endurance training in older healthy adults (67.23 ± 3.40 years). While the results showed a significant interaction effect in MTC variability, stride time, and stride length variability did not show such an effect. The authors assume that a capable central nervous system, which controls gait variables, tries to accurately control the endpoints (e.g., MTC) with a higher priority than less important variables (e.g., stride length or stride time) due to the potentially increased risk for a trip-related fall [compare (49, 50)].

The results of Lamoth et al. support the notion that gait is not merely an automated motor activity but required higher cognitive functions especially in the elderly (51). Moreover, Hamacher et al. report elevated parameters of gait variability in patients with chronic LBP under cognitive dual task (serial three subtractions while walking) but not in the healthy control group (52). The authors assumed that due to the pain-related sensory dysfunctions of patients with chronic LBP, the gait variability is increased. To compensate this dysfunction, higher cognitive functions are required to control posture. Limited cognitive abilities, in turn, could lead to insufficient compensation of sensory dysfunctions and increase the risk of falling. Therefore, exercise interventions to prevent falls in patients suffering of chronic LBP should also focus on improving cognitive functions. Remarkably, it has been speculated that a coordinative–cognitive training including motor–cognitive dual-task exercises is more effective in improving cognitive functioning than a single task (26, 29, 53). Therefore, the efficacy of a multicomponent exercise program in patients suffering from chronical LBP may be raised further by adding motor–cognitive dual-task exercises (54).

Overall, the effects were too small to be considered clinically significant especially looking at the short study period of this pilot study. However, these results indicate that the applied MI has the potential to improve parameters relevant to the individual fall risk in a more sustainable way than standard PT in patients with LSS, chronic LBP, and neurogenic claudication and/or degenerative spondylolisthesis.

In future studies, this multimodal approach should be applied to larger cohorts to consolidate the results. Furthermore, cognitive parameters such as central activation (e.g., with functional near-infrared spectroscopy) during simple/dual-task walking should be involved in the procedure, and other kinematic gait parameters should be taken into consideration. This would allow even better estimations about the extent of gait automaticity and thus about the effect on risk of falling of such a multimodal intervention.

## Data Availability Statement

The datasets generated for this study are available on request to the corresponding author.

## Ethics Statement

The studies involving human participants were reviewed and approved by Ethics committee of the Otto von Guericke University Magdeburg, Germany. The patients/participants provided their written informed consent to participate in this study.

## Author's Note

Over 500 million people worldwide suffer from chronic low-back pain and the associated health restrictions, regardless of gender or economic status. The effects of this impairment are not only physical loss of function, but are also perceived on a psychosocial level, resulting in reduced quality of life. The pain is often accompanied by insufficient muscular stabilization and poor posture. All these factors lead to a change in gait pattern, which may lead to an increased risk of falling. Nearly one third of people over 65 years of age fall every year and with advancing age the risk of falling increases significantly. People with chronic low-back pain have an even higher prevalence of falls. A purely biomedical addressing of the complaints is not suitable for many patients due to the manifold biosocial effects. Many patients seek relief from symptoms in alternative treatment strategies. Dance training integrating physical activity, multiple cognitive, and social elements in combination with additional coordinative-cognitive exercises as a multidisciplinary and multimodal approach aims exactly this complex problem. We expect reduced gait variability, improved mobility, and physical function leading to an increased quality of life and a decreased risk of falling in low-back pain patients.

## Author Contributions

K-CB: study execution and writing manuscript. TB: study execution, statistics, writing assistance, and proof reading. DH: study execution and proof reading. SB, TG, CC, KM, and AN: study execution. CS: study design and execution. JF and LS: study design, execution, and proof reading. All authors contributed to the article and approved the submitted version.

## Conflict of Interest

The authors declare that the research was conducted in the absence of any commercial or financial relationships that could be construed as a potential conflict of interest.
